# Absenteeism and Employer Costs Associated With Chronic Diseases and Health Risk Factors in the US Workforce

**DOI:** 10.5888/pcd13.150503

**Published:** 2016-10-06

**Authors:** Garrett R. Beeler Asay, Kakoli Roy, Jason E. Lang, Rebecca L. Payne, David H. Howard

**Affiliations:** Author Affiliations: Kakoli Roy, Rebecca L. Payne, Office of the Associate Director for Policy, Centers for Disease Control and Prevention, Atlanta, Georgia; Jason E. Lang, National Center for Chronic Disease Prevention and Health Promotion, Centers for Disease Control and Prevention, Atlanta, Georgia; David H. Howard, Rollins School of Public Health, Emory University, Atlanta, Georgia.

## Abstract

**Introduction:**

Employers may incur costs related to absenteeism among employees who have chronic diseases or unhealthy behaviors. We examined the association between employee absenteeism and 5 conditions: 3 risk factors (smoking, physical inactivity, and obesity) and 2 chronic diseases (hypertension and diabetes).

**Methods:**

We identified 5 chronic diseases or risk factors from 2 data sources: MarketScan Health Risk Assessment and the Medical Expenditure Panel Survey (MEPS). Absenteeism was measured as the number of workdays missed because of sickness or injury. We used zero-inflated Poisson regression to estimate excess absenteeism as the difference in the number of days missed from work by those who reported having a risk factor or chronic disease and those who did not. Covariates included demographics (eg, age, education, sex) and employment variables (eg, industry, union membership). We quantified absenteeism costs in 2011 and adjusted them to reflect growth in employment costs to 2015 dollars. Finally, we estimated absenteeism costs for a hypothetical small employer (100 employees) and a hypothetical large employer (1,000 employees).

**Results:**

Absenteeism estimates ranged from 1 to 2 days per individual per year depending on the risk factor or chronic disease. Except for the physical inactivity and obesity estimates, disease- and risk-factor–specific estimates were similar in MEPS and MarketScan. Absenteeism increased with the number of risk factors or diseases reported. Nationally, each risk factor or disease was associated with annual absenteeism costs greater than $2 billion. Absenteeism costs ranged from $16 to $81 (small employer) and $17 to $286 (large employer) per employee per year.

**Conclusion:**

Absenteeism costs associated with chronic diseases and health risk factors can be substantial. Employers may incur these costs through lower productivity, and employees could incur costs through lower wages.

## Introduction

Since the 1980s, the prevalence of chronic diseases and unhealthy lifestyle behaviors among American adults has increased. For instance, in 1980, the prevalence of hypertension was 24% and diabetes prevalence was below 3%; however, by 2012 the prevalence of hypertension was 29% and diabetes 9% (1–3). In the US working population, one-third of adults are obese (4,5), approximately 1 in 5 is a smoker (6), and more than half do not meet physical activity recommendations (4,7). The Centers for Disease Control and Prevention recognizes obesity, tobacco use, and physical inactivity as “winnable battles.” Winnable battles are public health priorities for which effective evidence-based interventions exist and could be broadly implemented to bring about large reductions in illness and death.

Employers are interested in reducing rates of chronic disease and reducing health risk factors because employers bear about 58% of total employee medical costs (8). Compared with their counterparts, employees who have chronic diseases and unhealthy lifestyle behaviors have higher medical costs, miss more workdays, and are potentially less productive at work (9–12). The increasing age of the labor force may increase the prevalence of these conditions and their associated costs (13). In light of these considerations, many employers have adopted workplace wellness programs to improve health-related behaviors and reduce the incidence of chronic conditions (14).

Numerous studies have quantified the effect of diseases and risk factors on absenteeism (9–12,15–17). These studies often focused on a single condition and used various data sets, samples, and time periods, making it difficult to estimate the benefits of policies that affect multiple conditions simultaneously. Among studies that analyzed multiple conditions, some did not separately estimate absenteeism or account for other employee characteristics that may confound the relationship between chronic conditions and missed workdays.

The objective of this study was to estimate the association between absenteeism and 5 conditions: 3 risk factors (obesity, smoking, physical inactivity) and 2 chronic diseases (hypertension and diabetes). We applied the same method and regression framework to all conditions and analyzed absenteeism in 2 of the most commonly used data sources for health care costs — health risk appraisals (HRAs) from a large privately insured claims database (MarketScan) and a nationally representative health expenditure survey (the Medical Expenditure Panel Survey [MEPS]).

## Methods

We analyzed absenteeism from 2008 through 2011 using samples of respondents who were aged 18 to 64 years, were continuously enrolled in the databases and employed for at least 1 year, were not pregnant, and responded to the question on missed workdays.

We analyzed data from the HRA subsample of the Truven Health MarketScan database. MarketScan is one of the largest commercial health care claims databases in the United States, representing more than 50 million employees and their dependents; the database is a de-identified convenience subpopulation of privately insured individuals (18). The HRA is a voluntary 3%-to-5% subsample of the MarketScan commercial claims data and contains self-reported information on health status (eg, hypertension) and health risk factors (eg, smoking). If a respondent completed more than 1 HRA in any year, we used the most recent HRA submitted.

MEPS is a publicly available, de-identified, annual survey consisting of a 2-year rolling panel design that collects data on the health care use and health status of respondents (19). The MEPS sample is representative of the civilian, noninstitutionalized US population. Our study used variables that were self-reported by respondents.

We measured absenteeism in MarketScan using responses to the question, “In the past 6 months how many days have you missed work due to illness or injury?” We annualized workdays missed by multiplying responses by 2. In MEPS we measured absenteeism using responses to the question, “How many times [did you] miss more than a half-day of work since the last interview?” We annualized the missed workday variable by adjusting responses for the length of time respondents met the inclusion criteria.

We classified MarketScan respondents as physically inactive if they reported exercising fewer than 4 to 5 times per week with at least 30 minutes of moderate activity per period of exercise (20). We classified MEPS respondents as physically inactive if they responded no to the question asking if they “spend half an hour or more in moderate or vigorous physical activity at least three times a week.” We classified respondents as obese if their body mass index (BMI in kg/m^2^) was reported as 30 or higher. BMI was self-reported in MEPS; we calculated BMI from self-reported height and weight in MarketScan as ([weight in pounds × 703]/height in inches^2^). We classified respondents as smokers if they responded affirmatively to the question, “Do you currently smoke?” MEPS respondents with diabetes and hypertension were identified according to responses to questions that asked them if they had “ever been told by a doctor or other health professional” that they had the disease. For MarketScan, respondents also self-reported hypertension or diabetes but did not distinguish between physician-diagnosed and self-diagnosed disease.

We adjusted for age categories (18–34, 35–49, 50–64 y), sex, educational attainment (no high school degree or GED [general educational development], high school degree, some college or college degree, or graduate degree), region (Northeast, Midwest, South, and or West), industry (11 classifications [Box]), and employment (full-time or part-time). MEPS data allowed for additional control factors: union membership (yes, no, inapplicable), race/ethnicity (non-Hispanic white, non-Hispanic black, Hispanic, Asian), occupation code of the employee (14 classifications [Box]), employer-paid sick leave (yes or no), and whether the respondent had private insurance (yes or no). We used an indicator for whether the respondent lived in a metropolitan statistical area (MSA) as a summary statistic to compare data sources but did not include MSA in regression analyses.

**Box. Industry Categories in MarketScan and Medical Expenditure Panel Survey**

**MarketScan**

Agriculture, forestry, fishing

Communications, utilities

Construction

Finance, insurance, real estate

Manufacturing, durable goods

Manufacturing, nondurable goods

Oil & gas extraction, mining

Retail trade

Services

Transportation

Wholesale

**Medical Expenditure Panel Survey**

Agriculture

Construction

Financial services

Fishing

Forestry

Manufacturing

Mining

Other services

Professional services

Real estate

Retail trade

Transportation

Utilities

Wholesale trade

### Statistical analysis

We analyzed both data sets using Stata 12.1 and estimated a zero-inflated Poisson regression model (21,22) where the dependent variable was the number of self-reported workdays missed. The zero-inflated Poisson model accounts for additional zeroes in the dependent variable (missed workdays) by first fitting a logistic regression to predict the likelihood of an excess zero and then fitting a Poisson regression to obtain the count of missed workdays for nonexcess zeroes. We used the same set of explanatory variables as controls for both stages of the regression analysis. To estimate excess absenteeism or excess missed workdays, we used Stata’s *margins* command to provide the predicted difference in missed workdays for those with and those without a risk factor or chronic disease. For the MarketScan data, we controlled for respondents who completed an HRA in more than 1 year with an indicator. We also included year indicators, clustered by respondent, and used robust standard errors in all regressions. All analyses using MEPS data were weighted to produce estimates of the civilian, noninstitutionalized population meeting the sample inclusion criteria. Standard errors were adjusted for the MEPS’s complex survey design.

We estimated 6 sets of regressions in 2 categories. First, we estimated the effect of each risk factor and chronic disease separately (smoking, obesity, physical inactivity, diabetes, and hypertension) while adjusting for different levels of controls (none, basic demographic, and full model). In doing so, we observed how estimates of absenteeism changed as we adjusted for additional factors. In the regressions, we adjusted for demographic variables (eg, age group, sex, and region), year of survey, work status indicators (eg, union membership, full-time status), and industry indicators (eg, industry type). In the second set of 3 regressions, we estimated risk factors and chronic diseases separately (ie, risk factors only and chronic diseases only). Finally, we estimated the association between the number of conditions, risk factors, chronic diseases, and total missed workdays. 

To compute the base cost of absenteeism for the employed workforce, we multiplied the estimated number of excess missed workdays per year by daily compensation costs for an 8-hour workday. We calculated compensation costs using the Bureau of Labor Statistics average hourly compensation cost for US employers in 2011 ($30.45) (23). We adjusted hourly employee compensation to 2015 dollars using the employment cost index ($33.00) (24).

An employee’s absence may reduce the productivity of coworkers, particularly when work relies on team production. To capture the team-based spillover effect of a missed workday, we multiplied absenteeism costs by 1.6 (11,15,25). In an alternate scenario and to derive our lower-bound estimate on cost, we allowed for the scenario where an employee was able to make up work, or when work was completed by colleagues. In this scenario we multiplied costs by 0.43 (26). Using these 2 scenarios, the lower- and upper-bound range for the cost of a 1-hour absence would be $14.19 and $52.81 (in 2015 dollars).

To obtain condition-specific absenteeism costs, we multiplied the cost per person per year by the prevalence of the condition. When computing national costs, we used the MEPS prevalence and absenteeism estimates and multiplied them by the total employed population in the United States in 2011: 139 million people (27).

To estimate costs for employers, we assumed a small employer had 100 employees and a large employer had 1,000 employees. Because MEPS is a nationally representative survey, we used the MEPS data to extrapolate costs for small employers. We used MarketScan estimates to extrapolate costs for large employers.

MarketScan HRA and MEPS are de-identified data sets; our research did not involve human subjects (defined by Title 45 Code of Federal Regulations, Part 46). Therefore, approval from an institutional review board was not required.

## Results

In MarketScan, 356,758 observations met our inclusion criteria (Table 1). Of these, 127,143 individuals filled out an HRA in more than 1 year; thus, the 356,758 observations came from 229,615 individuals. The prevalence rates of the 5 conditions studied in the MarketScan sample were 27.4% for current smoking, 55.9% for physical inactivity, 26.0% for obesity, 18.0% for hypertension, and 4.8% for diabetes. In the MEPS analysis, 24,006 individuals met inclusion criteria. The MEPS prevalence rates were 17.2% for current smoking, 39.7% for physical inactivity, 30.0% for obesity, 24.9% for hypertension, and 6.0% for diabetes. The average number of missed workdays was 2.2 days per year for all employees in the MarketScan sample and 2.8 days per year for all employees in the MEPS sample. In MarketScan, 200,665 (56.2%) respondents and in MEPS 12,498 (52.1%) respondents reported zero absenteeism days.

**Table 1:** Summary Statistics on US Workforce: Data From MarketScan^a^ and Medical Expenditure Panel Survey, 2008–2011^b^^,^ c

Variable	MarketScan (No. of Observations = 356,758^d^)	Medical Expenditure Panel Survey (N = 24,006)
**Self-reported workdays missed, n (SD)**	2.2 (12.6)	2.8 (8.7)
**Female**	129,503 (36.3)	10,075 (42.0)
**Age group, y**		
18–34	93,064 (26.1)	7,344 (30.6)
35–49	154,766 (43.4)	9,435 (39.3)
50–64	108,928 (30.5)	7,227 (30.1)
**Current smoking^e^**	60,477 (27.4)	4,122 (17.2)
**Former smoking**	12,126 (23.7)	NA
**Physical inactivity^e^**	28,568 (55.9)	9,539 (39.7)
**Obesity**	92,910 (26.0)	7,190 (30.0)
**Hypertension^e^**	35,750 (18.0)	5,972 (24.9)
**Diabetes^e^**	7,361 (4.8)	1,431 (6.0)
**Education^e^**		
No high school degree or GED	776 (0.4)	2,752 (11.5)
High school degree	11,740 (6.1)	10,229 (42.6)
Some college/college degree	178,120 (91.8)	5,654 (23.6)
Graduate degree	3,316 (1.7)	5,371 (22.4)
**Metropolitan statistical area**	341,029 (95.6)	20,470 (85.3)
**Region^f^**		
Northeast	104,504 (29.3)	4,308 (17.9)
Midwest^g^	38,541 (10.8)	5,368 (22.4)
South	118,979 (33.4)	8,954 (37.3)
West	94,563 (26.5)	5,376 (22.4)
**Part-time employee^h^**	3,157 (0.9)	NA
**Employer offers paid sick leave^i^**	NA	15,753 (65.6)
**Union membership**	30,3 (8.5)	2,886 (12.0)
**Private insurance^i^**	NA	20,389 (84.9)

Abbreviations: GED, general educational development; NA, not applicable.

a MarketScan is a large US commercial health care claims database; it is a de-identified convenience subpopulation of privately insured individuals (18).

b All values are number (percentage) unless otherwise indicated. Percentages may not total 100 because of rounding.

c All values are self-reported.

d The 356,758 observations were of 229,615 individuals.

e MarketScan denominators differed according to number of respondents to question.

f Northeast: Connecticut, Maine, Massachusetts, New Hampshire, New Jersey, New York, Pennsylvania, Rhode Island, Vermont. Midwest: Illinois, Indiana, Iowa, Kansas, Michigan, Minnesota, Missouri, Nebraska, North Dakota, Ohio, South Dakota, and Wisconsin. South: Alabama, Arkansas, Delaware, District of Columbia, Florida, Georgia, Kentucky, Louisiana, Maryland, Mississippi, North Carolina, Oklahoma, South Carolina, Tennessee, Texas Virginia, and West Virginia. West: Alaska, Arizona, California, Colorado, Hawaii, Idaho, Montana, Nevada, New Mexico, Oregon, Utah, Washington, and Wyoming. 171 observations had missing values for regions.

g MarketScan’s Midwest region is called “North Central.”

h The Medical Expenditure Panel Survey sample excluded part-time employees.

i MarketScan analysis did not identify those with paid sick leave; it used a sample of privately insured individuals.

**Figure 1:**
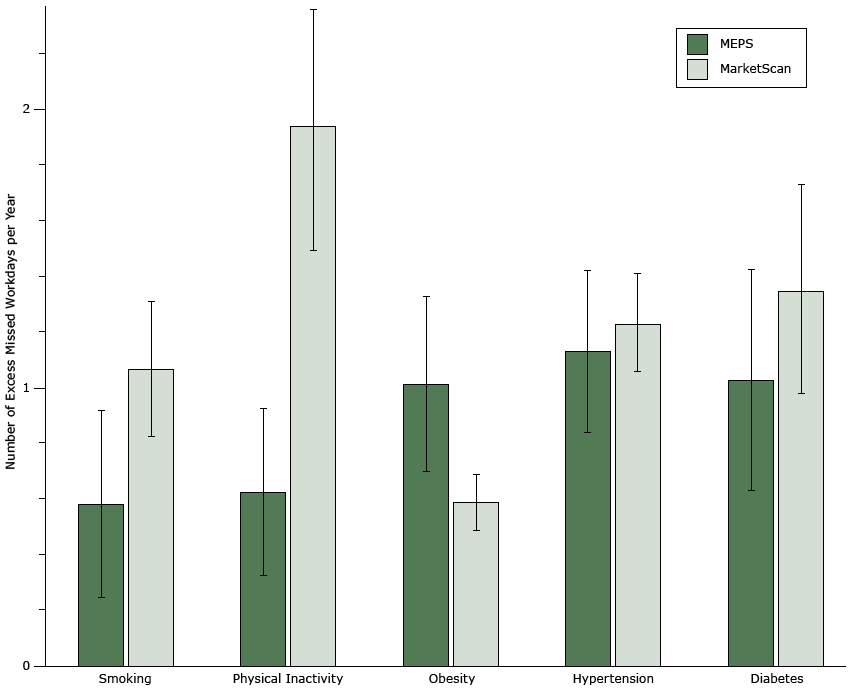
Regression-adjusted number of excess missed workdays per year by chronic disease or risk factor from the full model, which controlled for industry and employee characteristics. Error bars indicate 95% confidence intervals. Abbreviation: MEPS, Medical Expenditure Panel Survey. **Table:** 

MEPS and MarketScan estimates of the number of regression-adjusted excess missed workdays in the full model were qualitatively similar to each other, although the point estimates were different (Figure 1 and Table 2). MarketScan captures data on a more homogenous and potentially more economically advantaged population than does MEPS. Hence, it was not surprising that the results between the 2 data sets diverged slightly. However, as we adjusted for additional covariates the estimates for each data set became closer to each other in magnitude. Differences between the 2 data sets remained for estimates of physical inactivity and obesity.

Variable	No. of Excess Missed Workdays per Year (95% Confidence Interval)
Medical Expenditure Panel Survey	
Smoking	0.58 (0.25–0.92)
Physical inactivity	0.63 (0.33–0.92)
Obesity	1.02 (0.70–1.33)
Hypertension	1.13 (0.84–1.42)
Diabetes	1.03 (0.63–1.42)
MarketScan	
Smoking	1.07 (0.83–1.31)
Physical inactivity	1.94 (1.51–2.36)
Obesity	0.59 (0.49–0.69)
Hypertension	1.23 (1.06–1.41)
Diabetes	1.35 (0.98–1.73)

**Table 2:** Regression-Adjusted Estimates of the Effect of Selected Health Risk Factors or Chronic Diseases on Number of Excess Missed Workdays^a^ per Year per Employee in US Workforce^b^^,^ c

Risk Factor or Disease	Single Factor or Disease^d^			Multiple Factors or Diseases^e^		
	No Controls	Adjusted for Age, Sex, Race	Full Model	Risk Factor	Chronic Disease	All
**Medical Expenditure Panel Survey **						
Current smoking	0.67 (0.35–0.99)	0.76 (0.44–1.08)	0.58 (0.25–0.92)	0.66 (0.59–0.71)	NA	0.26 (0.19–0.31)
Physical inactivity	0.73 (0.45–1.01)	0.64 (0.35–0.93)	0.63 (0.33–0.92)	0.49 (0.44–0.52)	NA	0.50 (0.44–0.55)
Obesity	1.29 (0.98–1.60)	1.12 (0.79–1.44)	1.02 (0.70–1.33)	0.95 (0.90–1.00)	NA	0.20 (0.17–0.26)
Hypertension	1.51 (1.21–1.82)	1.28 (0.97–1.58)	1.13 (0.84–1.42)	NA	1.08 (1.03–1.12)	0.96 (0.90–1.00)
Diabetes	1.49 (1.09–1.88)	1.17 (0.77–1.57)	1.03 (0.63–1.42)	NA	0.72 (0.63–0.80)	0.62 (0.53–0.70)
**MarketScan**						
Current smoking	1.77 (1.64–1.90)	1.82 (1.69–1.97)	1.07 (0.83–1.31)	1.47 (0.07–2.2)	NA	1.51 (0.77–2.25)
Current smoking, controlled for former smoking	2.41 (1.66–3.16)	2.31 (1.57–3.05)	2.20 (1.45–2.95)	2.21 (1.76–2.66)	NA	1.95 (1.23–2.74)
Physical inactivity	2.23 (1.80–2.67)	2.06 (1.64–2.49)	1.94 (1.51–2.36)	1.70 (1.26–2.13)	NA	1.64 (1.20–2.08)
Obesity	0.92 (0.81–1.02)	0.75 (0.66–0.85)	0.59 (0.49–0.69)	1.16 (0.71–1.6)	NA	0.88 (0.42–1.34)
Hypertension	2.38 (2.20–2.55)	2.06 (1.64–2.31)	1.23 (1.06–1.41)	NA	0.73 (0.52–0.95)	0.85 (0.31–1.37)
Diabetes	2.29 (1.97–2.62)	2.05 (1.72–2.38)	1.35 (0.98–1.73)	NA	1.17 (0.82–1.53)	1.71 (0.77–2.65)

Abbreviations: MEPS, Medical Expenditure Panel Survey; NA, not applicable.

a Number of excess missed workdays defined as the difference in the number of days missed from work by those who reported having a risk factor or chronic disease and those who did not.

b Data sources: MarketScan and Medical Expenditure Panel Survey [19], 2008–2011. MarketScan is a large US commercial health care claims database; it is a de-identified convenience subpopulation of privately insured individuals (18).

c Regression estimates from zero-inflated Poisson regression, robust standard errors clustered by respondent (MarketScan). In addition, MEPS estimates weighted and standard errors adjusted for complex survey design.

d Models for 1 risk factor or chronic disease in regression (specified by the row). The column depicts level of controls where the full model includes controls for age, sex, race (MEPS only), industry, part-time status, union membership, region, and sick-leave policy (MEPS only).

e Models for multiple risk factors or diseases, listed by row (eg, risk factors regression includes smoking, inactivity, and obesity). All regressions include controls for age, sex, race (MEPS only), industry, part-time status, union membership, region, and sick-leave policy (MEPS only).

Individuals with a condition had significantly greater absenteeism than those without one. For example, using MarketScan we estimated that a smoker will miss 1.07 (95% confidence interval [CI], 0.83–1.31) more workdays than a nonsmoker. Across all conditions, as we adjusted for additional covariates, the estimated magnitude of excess missed workdays decreased (Table 2). When we examined individuals with multiple risk factors or diseases, the effect of any 1 condition remained significant after we adjusted for other factors. Similarly, adding risk factors and chronic diseases in the same regression changed the magnitude of the coefficients, but significance and coefficient sign remained the same.

**Figure 2:**
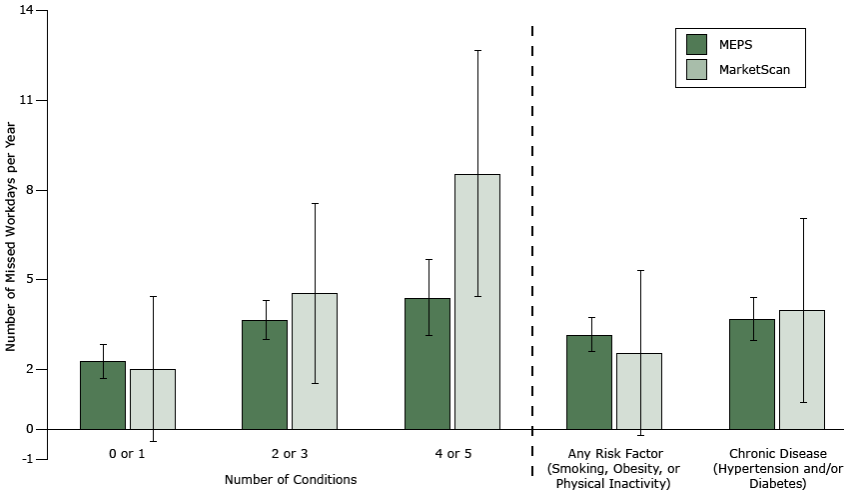
Regression-adjusted number of missed workdays per year. Error bars indicate 95% confidence intervals. Abbreviation: MEPS, Medical Expenditure Panel Survey. **Table:** 

We found a correlation between the number of conditions an employee reported and the predicted total number of missed workdays per year (Figure 2). Employees with 0 or 1 condition missed 2.0 workdays (MarketScan) or 2.3 workdays (MEPS). Employees with 2 or 3 conditions missed 4.5 days (MarketScan) or 3.7 days (MEPS). Finally, individuals with 4 or 5 conditions missed 8.6 days (MarketScan) or 4.4 days (MEPS). We found a similar pattern for number of risk factors or chronic diseases.

Survey Data Set	No. of Conditions			Any Risk Factor (Smoking, Obesity, or Physical Inactivity)	Chronic Disease (Hypertension or Diabetes)
	0 or 1	2 or 3	4 or 5		
MarketScan, no. of missed workdays	2.0 (−0.4 to 4.4)	4.5 (1.5 to 7.5)	8.6 (4.5 to 12.7)	2.6 (−0.2 to 5.4)	4.0 (0.9 to 7.1)
Medical Expenditure Panel Survey, no. of missed workdays	2.3 (1.7 to 2.9)	3.7 (3.0 to 4.4)	4.4 (3.1 to 5.7)	3.2 (2.6 to 3.8)	3.7 (3.0 to 4.4)

When we used our regression results to calculate the total cost of absenteeism to employers, we found that obesity had the highest total cost at $11.2 billion per year (Table 3). After obesity, total costs for each condition were ranked as follows: hypertension ($10.3 billion), physical inactivity ($9.1 billion), current smoking ($3.6 billion), and diabetes ($2.2 billion). In our sensitivity analyses, lower and upper estimates for costs ranged between $0.9 billion (diabetes) and $17.9 billion (obesity).

**Table 3:** Annual Cost of Absenteeism Borne by US Employers Because of Selected Health Risk Factors or Chronic Diseases Among Employees^a^

Risk Factor or Disease	Prevalence, %^b^	No. of People in US Workforce With Condition, in Millions^c^	No. of Excess Missed Workdays^d^ per Employee per Year^e^	Cost per Employee per Year, $^f^	Total US Cost per Year, Billions, $^g^	US Cost Scenario^h^, Lower–Upper, Billions, $
	*A*	*B*	*C*	*D*	*E*	*F*
Current smoking	17.0	23.6	0.58	153	3.6	1.6–5.9
Physical inactivity	39.7	55.2	0.63	166	9.1	3.9–14.6
Obesity	30.0	41.6	1.02	269	11.2	4.8–17.9
Hypertension	24.9	34.6	1.13	298	10.3	4.4–16.5
Diabetes	6.0	8.3	1.03	272	2.2	0.9–3.5

Abbreviation: MEPS, Medical Panel Expenditure Survey

a Data sources: MarketScan and Medical Expenditure Panel Survey [19], 2008–2011. MarketScan is a large US commercial health care claims database; it is a de-identified convenience subpopulation of privately insured individuals (18).

b Prevalence estimate from MEPS data (*A*).

c Assumes workforce population of 139 million people: *B* = *A* × 139 million.

d Number of excess missed workdays defined as the difference in the number of days missed from work by those who reported having a risk factor or chronic disease and those who did not.

e *C* = MEPS regression estimates for single factor or disease, full model (Table 2).

f Assumes an average employment cost of $33.00 per hour and an 8-hour work day (in 2015 dollars): *D* = *C* × 8 × 33.

g Total cost (*E*) = *B* × *D* in 2015 dollars.

h Lower and upper cost scenarios based on multipliers of 0.43 and 1.6 respectively: lower value = 0.43 × *E*; upper value = 1.6 × *E*.

Extrapolating these results to reflect employer size, we found that per-year absenteeism for a small employer ranged from 6 days for diabetes to 31 days for obesity and costs ranged from $1,621 for diabetes to $8,065 for obesity (Table 4). A large employer (1,000 employees) could face absenteeism rates of 65 days for diabetes to 1,083 days for physically inactive employees. Annual costs for a large employer could range from approximately $17,000 for diabetes to more than $285,000 for physical inactivity.

**Table 4:** Annual Total Absenteeism and Costs to Employers in the United States Because of Selected Health Risk Factors or Chronic Diseases Among Employees, by Size of Employer^a^

Condition	Prevalence, %	No. of Excess Missed Workdays^b^ per Employee	Total No. of Missed Workdays per Year per Employer	Total Cost per Year, $^c^	Cost per Employee per Year, $^c^
**Small firm (100 employees)^d^**					
Current smoking	17	0.58	10	2,603	26
Physical inactivity	40	0.63	25	6,603	66
Obesity	30	1.02	31	8,065	81
Hypertension	25	1.13	28	7,419	74
Diabetes	6	1.03	6	1,621	16
**Large employer (1,000 employees)^e^**					
Current smoking	27	1.07	292	77,117	77
Physical inactivity	56	1.94	1,083	285,785	286
Obesity	26	0.59	153	40,498	40
Hypertension	18	1.23	221	58,450	58
Diabetes	5	1.35	65	17,107	17

a Data sources: MarketScan and Medical Expenditure Panel Survey (19), 2008–2011. MarketScan is a large US commercial health care claims database; it is a de-identified convenience subpopulation of privately insured individuals (18).

b Number of excess missed workdays defined as the difference in the number of days missed from work by those who reported having a risk factor or chronic disease and those who did not.

c Based on average employment cost of $33.00 per hour and an 8-hour workday. All costs are in 2015 dollars.

d Small employer costs based on Medical Expenditure Panel Survey prevalence and estimated absenteeism.

e Large employer costs based on MarketScan prevalence and estimated absenteeism.

## Discussion

We estimated that absenteeism costs associated with each of the 5 conditions — hypertension, diabetes, smoking, physical inactivity, and obesity — were greater than $2 billion per condition per year. Accounting for costs imposed by absenteeism will be useful in assessing the impact of programs and policies that affect the prevalence of these conditions. We cannot determine from these data whether employers bear this cost through lower profits or employees bear the cost through lower wages.

After adjusting for wage growth to 2015, our estimates of absenteeism costs were similar to previous estimates: for instance, for obesity, our estimated cost of $11.2 billion (range, $4.8–$17.9 billion) was within the range of prior estimates: $4.3 billion in 2004 ($5.5 billion in 2015 dollars) and $12.8 billion in 2008 ($14.6 billion in 2015 dollars) (10,28). Regarding the magnitude of excess workdays missed, our estimate for smoking of about 1 day was smaller than reported previously (2.3–2.6 days) (12,16). However, when we added a control for former smokers, our MarketScan absenteeism estimate increased to 2.2 days. (MEPS did not report whether respondents were former smokers.)

Our absenteeism estimates for diabetes, 1 to 2 days, were smaller than estimates of previous studies (3 days) (9). Likewise, our physical inactivity estimates (1–2 days) were smaller than previous estimates (4 days) (17). Differences between our estimates and prior estimates may be attributable to our more recent study period and our inclusion of additional controls, which tend to reduce the number of missed workdays attributable to risk factors or diseases.

Our study has several limitations. First, health-related conditions (smoking, physical inactivity, obesity) are primary risk factors for chronic diseases such as hypertension and diabetes. We did not model the disease pathway or the potential interaction of health risk factors and chronic diseases; prior research has the same limitation. In addition, because we were not able to control for former smokers in the MEPS sample, we underestimated the number of missed workdays associated with smoking. Second, our employer-specific estimates were extrapolations; we did not have actual employer size as a variable in the data set. Third, we did not account for the relationship between the labor market (eg, employment and wages) and disease status. One potential labor market outcome could be that people with chronic conditions have lower-paying jobs. However, recent research found no difference in wages after controlling for health insurance costs (29). Fourth, our data did not allow us to distinguish between type 1 and type 2 diabetes; nevertheless, most (>95%) Americans who report having diabetes have type 2 diabetics, and our data probably reflects this proportion (30). Fifth, the MarketScan data are not nationally representative; however, MarketScan commercial encounters data in 2011 had more than 52 million enrollees (employees and dependents) and the database is projectable to 58% of the US population. The HRA sample is a voluntary subset of employees, and respondents may differ from the larger pool of MarketScan individuals in ways that reduce the generalizability of results.

This is the first study to examine absenteeism associated with multiple risk factors and chronic diseases using MarketScan and MEPS. We found the estimates of absenteeism to be similar for the 2 data sets, except for physical inactivity and obesity. The substantial difference in physical inactivity estimates may reflect differences between MarketScan and MEPS in how the question on physical activity was worded. We find robust evidence that risk factors and chronic diseases are strongly associated with increased employee absenteeism.

Workplace wellness programs have potential to reduce both medical and absenteeism costs (31). Although such programs, their comprehensiveness, and their potential returns vary, workplace programs are important partners in improving health. As an example, the American Heart Association reiterated in 2015 its commitment to workplace wellness as a way of improving cardiovascular health (14). While improving employee health and reducing absenteeism initially benefit employers and employees, a healthy workforce and an increase in productivity are national resources with benefits that extend beyond private sector employers and their employees.
